# West Midlands region less than full time training survey

**DOI:** 10.1192/bjo.2021.423

**Published:** 2021-06-18

**Authors:** Eleanor Parkinson, Fiona Hynes

**Affiliations:** Birmingham and Solihull Mental Health Foundation Trust

## Abstract

**Aims:**

To more fully understand the training experience of less than full time (LTFT) trainees working in psychiatry in the West Midlands Region with the aim of identifying areas that would improve the training experience.

**Background:**

LTFT training has grown in popularity since its formal introduction in 2007. The greater participation of women in medicine and generational changes in lifestyle expectations are some of the factors behind this trend. Approximately 13% of psychiatry trainees in the UK are training LTFT, bringing the benefit of allowing trainees to balance caring responsibilities or health conditions with continuing their postgraduate training. However it is not without its challenges for trainees which we aimed to explore in this survey.

**Method:**

An electronic survey was sent out to all trainees via email, LTFT trainees of all training grades were invited to respond. Trainees were contacted in the five mental health trusts making up the region. The survey contained 32 questions that covered a range of topics including educational opportunities, perceived attitudes to LTFT trainees and training experience. Data were collected over a six month period in 2019. There were 22 responses to the survey region-wide.

**Result:**

86% of respondents were working reduced sessions in full-time posts with implications for their clinical workload and 14% responded that their clinical contact time was not adjusted to reflect their working hours. 36% of respondents experienced difficulties attending their formal teaching programme while 82% had attended educational commitments on non-working days. 14% of respondents felt training LTFT did not allow them to meet training requirements while 23% would not recommend LTFT training in the West Midlands to others. Trainees cited difficulties managing a full time workload and not having support from supervisors as reasons for these views. 40% of respondents reported experiencing negative attitudes from seniors and 50% felt isolated from other trainees due to LTFT training status.

**Conclusion:**

The survey has developed our understanding of the challenges faced by LTFT trainees and it has been communicated regionally and to employing trusts to promote action. For example, at a trust level, the use of personalised work schedules can address some common difficulties. More effectively communicating sources of support to trainees, sharing best practice and providing networking opportunities are suggested as next steps regionally. New administrative processes to maintain an accurate list of LTFT trainees is vital in implementing this. Improving the information given to trainers is another development area.

